# Graphite Based Electrode for ECG Monitoring: Evaluation under Freshwater and Saltwater Conditions

**DOI:** 10.3390/s16040542

**Published:** 2016-04-15

**Authors:** Tharoeun Thap, Kwon-Ha Yoon, Jinseok Lee

**Affiliations:** 1 Department of Biomedical Engineering, Wonkwang University School of Medicine, 460 Iksandeaero, Iksan, Jeonbuk 570-749, Korea; bami1314@wku.ac.kr; 2 Department of Radiology, Wonkwang University School of Medicine, 460 Iksandeaero, Iksan, Jeonbuk 570-749, Korea

**Keywords:** graphite, pencil lead, carbon, ECG electrode, dry electrode, ECG monitoring, underwater, freshwater, saltwater

## Abstract

We proposed new electrodes that are applicable for electrocardiogram (ECG) monitoring under freshwater- and saltwater-immersion conditions. Our proposed electrodes are made of graphite pencil lead (GPL), a general-purpose writing pencil. We have fabricated two types of electrode: a pencil lead solid type (PLS) electrode and a pencil lead powder type (PLP) electrode. In order to assess the qualities of the PLS and PLP electrodes, we compared their performance with that of a commercial Ag/AgCl electrode, under a total of seven different conditions: dry, freshwater immersion with/without movement, post-freshwater wet condition, saltwater immersion with/without movement, and post-saltwater wet condition. In both dry and post-freshwater wet conditions, all ECG-recorded PQRST waves were clearly discernible, with all types of electrodes, Ag/AgCl, PLS, and PLP. On the other hand, under the freshwater- and saltwater-immersion conditions with/without movement, as well as post-saltwater wet conditions, we found that the proposed PLS and PLP electrodes provided better ECG waveform quality, with significant statistical differences compared with the quality provided by Ag/AgCl electrodes.

## 1. Introduction

One of the most important methods of monitoring vital signs that track an individual’s health is using an electrocardiogram (ECG or EKG). It provides meaningful information regarding person’s heart performance and functionality. ECG is a recording of the electrical activity of the myocardium during one cardiac cycle. It is characterized by a recurrent sequence of PQRST waves and a conditional U-wave, and it is recorded by placing electrodes on a body surface in a non-invasive manner [1]. Each different ECG wave signature correlates with a phase of the heart’s activity, and the observed characteristics are clinically important in diagnostic decision for various cardiac diseases [2]. The information extracted from the morphological changes in the ECG waveform can be used to diagnose cardiac ischemia, injury, and malignant arrhythmias [3,4]. The cardiovascular system, the heart and circulation, are mostly controlled by higher brain centers and cardiovascular control areas in the brain through the sympathetic and parasympathetic branches of the autonomic nervous system. Efferent sympathetic and vagal activities directed to the sinus node are characterized by discharges, largely synchronous with each cardiac cycle, which can be modulated by central and peripheral oscillators. These mechanisms generate rhythmic fluctuations in efferent neural discharge, which manifest short and long-term oscillations in the heart period. Heart rate variability (HRV) revealed by ECG analysis becomes a non-invasive technique for assessing autonomic influences on the heart [5,6,7,8].

Based on various clinical needs, much effort has been spent lately to make ECG monitoring an easy and “anywhere-anytime” available procedure [9,10,11,12,13,14,15,16,17,18]. However, ECGs can be measured and analyzed in a limited variety of environments. Especially in the water-submerged condition, the signal quality is dramatically decreased; thus, the ECG signal analysis such as HRV becomes highly debatable. In underwater environments, pressure, temperatures, and depth have an impact on the human physiological control systems [19]. As the control systems break down due to prolonged hyperbaric exposures, humans are more susceptible to detrimental conditions such as hypothermia, hypoxia, and many neurological and cardiovascular problems including decompression sickness (DCS) [20,21,22,23]. It is reported that early detection of DCS can be made from HRV analysis [23,24].

Underwater ECG monitoring further can be extended to life vest equipment and aqua therapy with the main purpose to ensure safety of any water-related activity. A fully functional and reliable underwater ECG monitoring system, capable of recording ECG waveforms, does not currently exist to support either shallow or deep diving medical research. ECG electrodes that can operate in water-submerged environments have just been recently developed [14]. It is reported that the Ag/AgCl electrode was replaced by carbon black powder to obtain ECG signals underwater without the use of waterproof adhesive tape. However, there are no published studies yet that have analyzed the performance under saltwater immersion.

Graphite is an allotrope of carbon and is a substance made of pure carbon. Other allotropes include diamonds, amorphous carbon, and charcoal. Graphite is mostly consumed for refractories, batteries, steelmaking, expanded graphite, brake linings, foundry facings, and lubricants. Graphite is also used as a pigment and a molding agent in glass manufacturing. Nuclear reactors also use graphite as an electron moderator. The detailed properties and characteristics of graphite have been specifically described elsewhere [25,26,27]. In this study, we evaluated graphite pencil lead (GPL) for ECG measurement under fresh- and saltwater. GPL is easily found and is widely used for writing and drawing. We have developed two types of dry electrodes from GPL and evaluated the usability of our electrodes in the seven different conditions: dry condition, freshwater immersion with/without movement condition, post-freshwater condition (*i.e.*, freshwater wet condition), saltwater immersion with/without movement condition, and post-saltwater condition (*i.e.*, saltwater wet condition). We have also described the fabrication process, the impedance characteristics, and the quality evaluation of the ECG morphology in each condition.

## 2. Materials and Methods

### 2.1. Fabrication of GPL Electrodes

**Figure 1:**
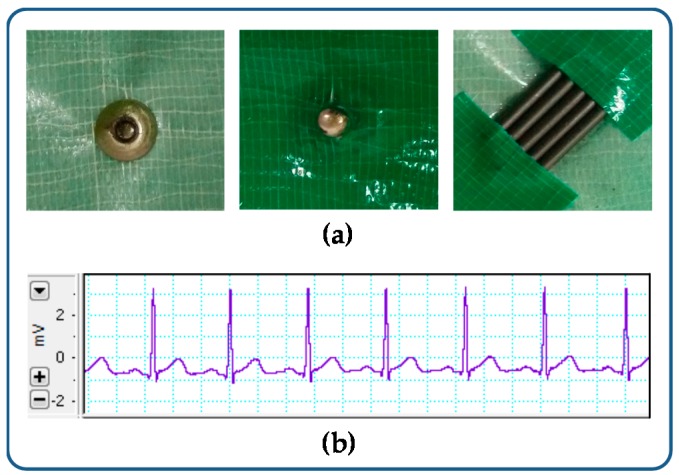
Pre-experiment for testing the usability of a graphite pencil lead electrode. (**a**) GPL on the metal cap; (**b**) Resultant signal.

In a pre-experiment, we simply cut and placed the GPL on the metal cap, and then held them altogether with duct tape as shown in Figure 1a. We connected the GPL with the PowerLab 8/35 and DualBioAmps FE135 (ADInstrument, Sydney, Australia), and contiguously placed for a standard 1-channel ECG monitor with sampling rate of 1 kHz and low pass filter cutoff frequency at 50 Hz. The resultant signal is shown in Figure 1b. With this evidence, we fabricated two types of GPL electrodes: pencil lead solid type (PLS) and pencil lead powder type (PLP).

#### 2.1.1. Pencil Lead Solid-Type (PLS) Electrode

**Figure 2:**
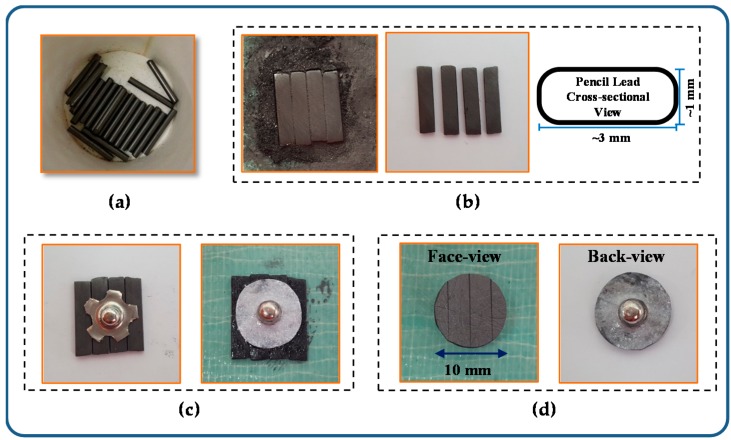
(**a**) Pencil leads; (**b**) Flattened pencil leads; (**c**) Each lead and the metal cap attached using a bond and then supported with a piece of paper; and (**d**) Pencil lead solid-type electrode.

PLS electrodes were fabricated as presented below:

(1) The 4B pencil leads were used as a material for making PLS electrodes. The pencil leads were cut into pieces as shown in Figure 2a.
(2) The cutting leads were flattened to form a rounded rectangle cross-sectional shape as shown in Figure 2b.
(3) The flattened pencil leads were arranged in a parallel manner with the metal cap placed on top and attached to each other by using an ethyl cyanoacrylate bond. The cap and lead surface are perfectly attached and conducted without bond infiltration as shown in Figure 2c. We applied the bond again around the metal cap and placed a piece of paper on it to support the pencil leads and cap.
(4) We circularly trimmed the edge. Figure 2d shows the face and back view of the PLS electrode.

For the performance evaluation, we fabricated the PLS electrode with a diameter of 10 mm and a thickness of 1 mm, which is the same size as the commercial Ag/AgCl electrode (3M Health Care, 2223H, Seoul, Korea).

#### 2.1.2. Pencil Lead Powder-Type (PLP) Electrode

**Figure 3:**
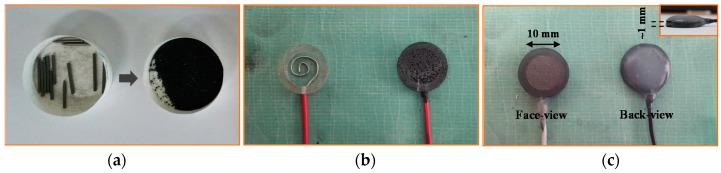
(**a**) Pencil lead grinding powder; (**b**) small piece of acrylic tube within electrical wire being placed at the bottom; and (**c**) pencil lead powder-type electrode.

PLP electrodes were fabricated as presented below:

(1) As same as PLS type, the 4B pencil leads were used as raw materials, and the pencil leads were peeled and grinded into powder as shown in Figure 3a.
(2) We mixed the pencil lead powder with chloroprene-rubber based bond and poured it into a piece of cylindrical acrylic tube within an electrical wire being placed at the bottom as shown in Figure 3b.
(3) We finally sanded the face of the electrode, made it smooth and flat, and subsequently applied the hot melted glue to support the wire on the backside as shown in Figure 3c.

**Figure 4:**
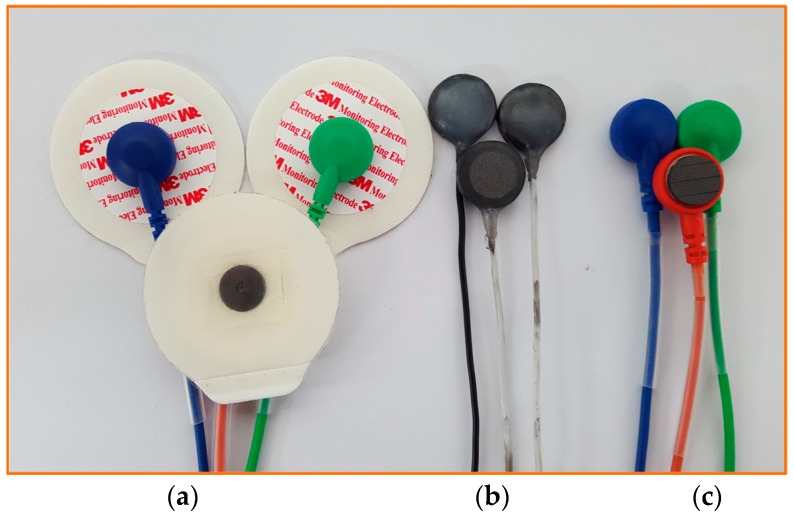
Three types of electrodes being used to perform the experiment: (**a**) commercial Ag/AgCl electrodes; (**b**) pencil lead powder-type electrodes; and (**c**) pencil lead solid-type electrodes.

In order to evaluate its performance, we also fabricated the PLP electrode with a diameter of 10 mm and thickness of 1 mm in order to compare it with the PLS and the commercial available Ag/AgCl electrode. Figure 4 shows three types of electrodes that we evaluated and used to compare the signal quality: the commercial Ag/AgCl electrodes (3M Health Care), PLP electrodes, and PLS electrodes.

### 2.2. Measurement Protocol and Signal Processing

In this study, twelve volunteer healthy subjects with ages ranging from 23 to 35 years (27.17 ± 3.33; mean ± STD), with average weight 74.75 ± 10.50 kg, and average height 173.33 ± 3.26 cm were included. The study protocol and data analysis were approved by the Institutional Review Board (IRB) at Wonkwang University of Hospital (WKUH201401-CTDV-001) and all volunteers consented to be subjects for the experiment.

The PowerLab 8/35 and DualBioAmps FE135 (ADInstrument) were simultaneously used to acquire ECG signals from the subjects. The ECG signals were displayed and recorded by using LabChart software (Ver. 7.3.7 Professional). We selected a sampling rate of 1 kHz with low pass filter cutoff frequency at 50 Hz for the frequency content of a QRS complex, which extends up to 40 Hz [28,29,30,31]. A minimal sampling rate of 500 Hz with a resolution of 12 bits has been recommended for the adult ECG by the American Heart Association [32,33,34], but for pediatric ECGs, higher sampling rates have been suggested [35,36]. Practically, for portable devices (e.g., Holter systems), the tendency is to reduce the sampling rate in order to increase the speed of analysis and to limit the data storage [37,38]. Thus, the sampling rate can be set up in the range between 100 and 1000 Hz in considering speed of analysis, data storage and time-resolution requirement [38,39,40,41]. In this paper, we used a high sampling rate of 1 kHz for the sophisticated evaluation of our proposed electrodes, which provides high time-resolution of 1 ms in analysis of HRV or waveform pattern. The recorded ECG signals were analyzed for R-peak detection and heart rate variability (HRV) using Matlab (R2012b).

ECG signals were simultaneously acquired and compared as follows: (Experiment-I) Ag/AgCl electrodes *vs.* PLS electrodes, and (Experiment-II) Ag/AgCl electrodes *vs.* PLP electrodes. As described in Table 1, each experiment was performed under two conditions, freshwater and saltwater. When all the experiments under freshwater were completed, the pool was washed and refilled with 400 liters of clean water mixed with 14 kg of coarse salt in order to make salt concentration similar to seawater (*i.e.*, on average 35 g/kg) [42]. The electrodes were contiguously placed for a standard 1-channel ECG by two trained cardiologists in Wonkwang University Hospital. For the electrode attachment on the body, the attachment skin sites were cleaned with 83% ethanol. The PLS and PLP electrodes were simultaneously attached with a regular surgical tape (3M Micropore surgical tape 1530S-1). We add that the PLS and PLP electrodes were reused throughout the week when data were collected, while new Ag/AgCl electrodes were provided to every subject for each measurement.

**Figure 5:**
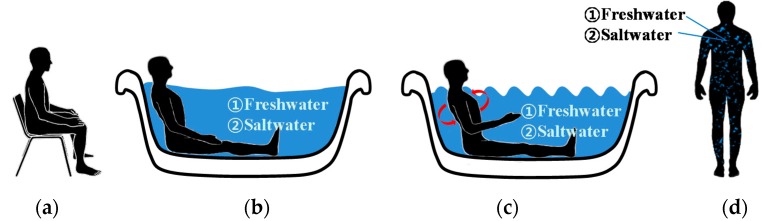
Experimental protocol for electrocardiogram data acquisition: (**a**) dry condition; Immersed condition (**b**) without movement; and (**c**) Side-to-side movement; and (**d**) wet condition.

Figure 5 summarizes all the steps, including Experiment I and II under freshwater and saltwater, respectively. Each experiment was performed for a total of 31 min. Each step was defined as follows:

Step 1: Five minutes in a sitting position outside the pool (the dry condition).
Step 2: Five-minute relaxation (lying position) inside the water pool (the immersion condition without movement).
Step 3: Three-minute side-to-side moving condition inside the water pool (the immersion condition with movement).
Step 4: Five minutes in the standing position outside the water pool (the wet condition).

The pool water temperature was 20.50 ± 1.30 °C; room temperature, 25 °C; and the outdoor temperature, 4.0 ± 1.0 °C. During the 5-min dry condition (Step 1), the subjects were asked to remain relaxed in the sitting position outside the pool; the electrodes at this point were completely dry. Step 1 is similar to a regular ECG measurement in a clinical environment. During the 5-min water-immersed condition (Step 2), the subjects were asked to immerse in the water pool, with water coming up to their neck so that all electrodes were fully immersed. Subsequently, the subjects were asked to perform side-to-side turning movements with the speed about 2 turns per second for 3 min (Step 3). Finally, the subjects were asked to exit the pool and remain relaxed in the standing position with the wet electrodes still applied to their skin for 5 min (Step 4).

The acquired ECG data were automatically filtered in the LabChart software (Ver.7.3.7 Pro.) through lowpass filter with cutoff frequency at 50 Hz. The signals were saved as Matlab file for data analysis. R-wave peak detection was performed on each recorded 31 minute-ECG signal by incorporating a filter bank with variable cut-off frequencies, spectral estimates of the heart rate, rank-order nonlinear filters and direct analysis [28,43]. The resultant R-wave peaks were confirmed by two trained cardiologists. For the HRV analysis, both time domain and frequency domain analysis were performed. We used HRV analysis as a quantitative performance measure since most previous hyperbaric studies [21,23,24,44] were concerned with changes in the dynamics of the autonomic nervous system.

HRV was measured in both time and frequency domains [45]. Time domain analysis included mean normal-to-normal (NN) interval (meanNN), standard deviation of all NN intervals (SDNN), the root mean square of the differences between adjacent NN intervals (RMSSD) and number of pairs of adjacent NN intervals differing by more than 50 ms in the entire recording (NN50). Frequency domain HRV analysis included low frequency (LF, 0.04–0.15 Hz), high frequency (HF, 0.15–0.4 Hz) bands, total power, and HF/LF ratio.

In the frequency domain analysis, we used RRI series along the time axis and analyzed with the fast Fourier transform (FFT). The analysis was performed using RRI time series, then the FFT yields complex numbers spaced evenly along the frequency axis from zero to half the sampling rate. The squared magnitude of each of the complex values from the FFT was then computed (real part squared plus imaginary part squared), as this yields the power spectral density (PSD) function. For both time and frequency domain analysis, premature ventricular contractions (PVCs) and premature atrial contractions (PACs) have a particularly insidious effect on each result since the effect of a sudden, brief change in RR interval (or HR) adds substantial power density to all frequencies or increases the statistical values, often overwhelming the true variability that is assessed [46]. On the other hand, if the RR intervals corresponding to the ectopic beats are simply deleted, individual frequency components change because the deletion of time advances the phase. This has the effect of reducing the calculated power density at some frequency components and augmenting it at others. We replaced the RR intervals affected by an ectopic beat with the same number of RR intervals of the value equal to the mean of those replaced.

For further performance analysis, we used cross-correlation coefficient measures. The ECG template obtained from Ag/AgCl electrode in the dry condition as a reference was used to quantify the signal quality obtained from PLS, PLP, and Ag/AgCl electrodes in the dry, fresh- and saltwater immersion and wet conditions by measuring cross-correlation coefficients *r* as defined by

(1) $$r=\frac{\sum_{i=1}^{N}X_{i}\cdot Y_{i}}{\sqrt{\sum_{i=1}^{N}X_{i}{}^{2}\cdot \sum_{i=1}^{N}Y_{i}{}^{2}}}$$

where $X_{i}$ represents the one cardiac cycle of ECG template obtained with the Ag/AgCl electrodes in the dry condition as a reference, which is clinically measured, and $Y_{i}$ represents the one cardiac cycle of ECG samples with each different electrode in each different condition. We calculated the cross-correlation coefficient values in the entire measured ECG samples.

Temporal and spectral measures of HRV and cross-correlation coefficients obtained from PLS and PLP electrodes were compared with those obtained from Ag/AgCl electrodes using the *t*-test. Statistical significance was accepted at *p* < 0.05.

### 2.3. Impedance Characterization

The electrode-to-skin contact impedances were analyzed for the three pairs of electrodes: PLS, PLP, and Ag/AgCl. The impedance was measured by using an impedance analyzer (Agilent 4294A precision impedance analyzer), which covers a broader test-frequency range from 40 Hz to 110 MHz, with a basic impedance accuracy of ±0.08%. The test signal level was 600 μA, and the frequency ranged from 40 Hz to 1 MHz. For each measurement, each pair of electrodes was placed on anterior forearm skin, 5 cm apart from each other. While ECG-power is mostly below 40 Hz; the electrodes for impedance measurements were chosen so that our results can be compared with the previous studies [11,47]. An elastic compression bandage was used to fix them to the skin. Prior to each measurement, the skin area was cleaned with a skin cleanser. For each of the subjects, all measurements were performed on the same day.

## 3. Results and Discussion

### 3.1. Dry and under Freshwater Condition (Case-I): PLS vs. Ag/AgCl and PLP vs. Ag/AgCl

**Figure 6:**
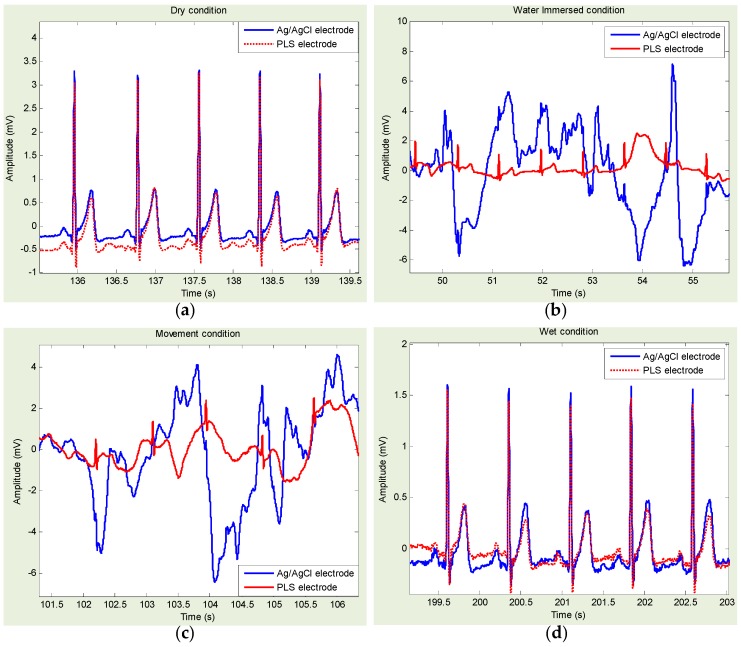
(Experiment I—Pencil lead solid-type (PLS) electrode compared with Ag/AgCl electrode) Example of electrocardiogram (ECG) signals for each experimental condition: (**a**) dry condition; freshwater immersed condition (**b**) without movement; and (**c**) with movement; and (**d**) freshwater wet condition. ECG signals acquired with Ag/AgCl (blue line) and PLS electrodes (red line).

Figure 6 shows approximately 5-s ECG data measurements using PLS and Ag/AgCl electrodes for each step: dry, freshwater immersion with/without movement, and freshwater wet condition. It is shown that the PLS electrodes provided all morphological components of ECG signals, in all conditions. Especially in freshwater immersion, with/without movement conditions, the ECG signal from PLS provided better quality than the one from Ag/AgCl, which is almost indiscernible.

**Figure 7:**
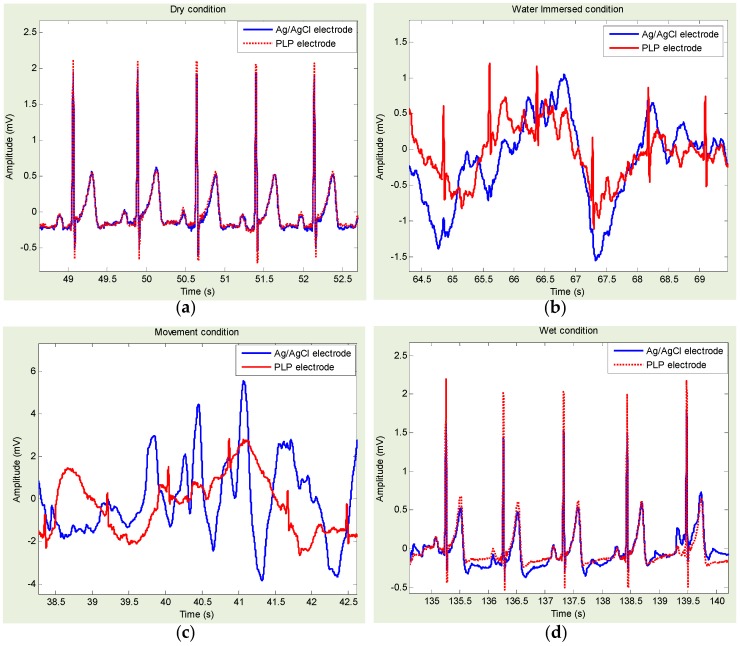
(Experiment II—Pencil lead powder-type (PLP) electrode compared with Ag/AgCl electrode) Example of electrocardiogram (ECG) signals for each experimental condition: (**a**) dry condition; freshwater immersed condition (**b**) without movement; and (**c**) with movement; and (**d**) freshwater wet condition. ECG signals acquired with Ag/AgCl (blue line) and PLP electrodes (red line).

Figure 7 shows approximately 5-s ECG data measurements using PLP and Ag/AgCl electrodes for each step: dry, freshwater immersion with/without movement, and freshwater wet condition. It is also shown that the PLP electrodes provided all morphological components of ECG signals, in all conditions. Similar to PLS, in freshwater immersion with/without movement conditions, the ECG signal from PLP provided better quality than the one from Ag/AgCl, which is almost indiscernible.

**Figure 8:**
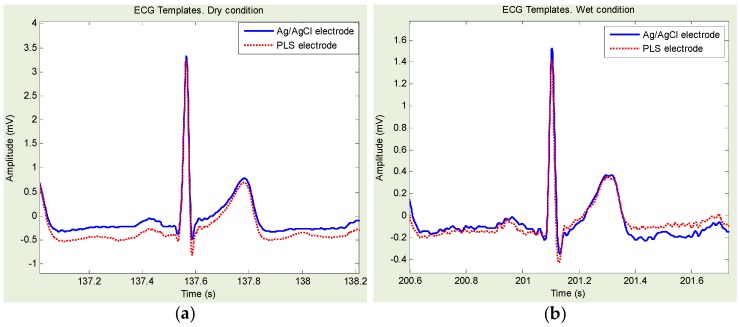
Example of electrocardiogram templates for dry and freshwater wet condition: (**a**) template derived from Figure 6a; and (**b**) template derived from Figure 6d.

**Figure 9:**
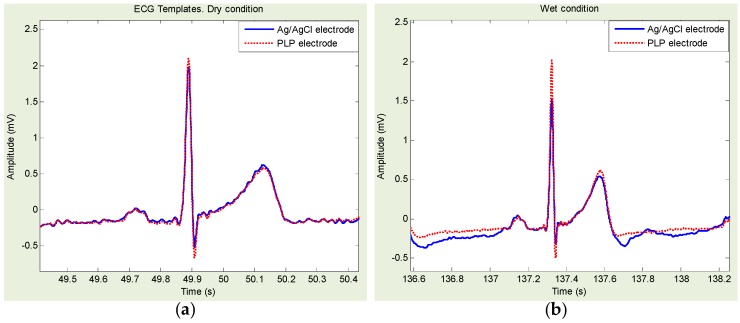
Example of electrocardiogram templates for dry and freshwater wet condition: (**a**) template derived from Figure 7a; and (**b**) template derived from Figure 7d.

Figure 8 shows an example of Ag/AgCl- and PLS-based ECG template directly derived from Figure 6a,d. Similarly, Figure 9 shows an example of Ag/AgCl- and PLP-based ECG template directly derived from Figure 7a,d. Ag/AgCl electrodes were good for dry and wet conditions. However, for the freshwater immersion with/without movement condition, the signals were hardly recognized, and the R-wave was almost indiscernible.

Statistical results of HRV indices and cross-correlation coefficients obtained with Ag/AgCl, PLS, and PLP electrodes are summarized in Table 2, Table 3, Table 4 and Table 5, for the dry, freshwater immersion without/with movement, and freshwater wet conditions, respectively. Regarding the temporal and spectral measures of HRV, under the dry and wet conditions, no statistical differences (*p* > 0.05) were identified for any of the temporal or spectral measures of HRV obtained within each pair: Ag/AgCl *vs.* PLS and Ag/AgCl *vs.* PLP. However, in the freshwater immersion conditions, we found significant statistical differences. More specifically, without movement, the statistical differences were identified when the Ag/AgCl *vs.* PLS was with SDNN, RMSSD, LF, and HF, the Ag/AgCl *vs.* PLP was with SDNN, RMSSD, and LF/HF as summarized in Table 3. With movement, the statistical differences identified when the Ag/AgCl *vs.* PLS was with mean NN, SDNN, RMSSD, and NN50, and the Ag/AgCl *vs.* PLP was with mean NN, SDNN, RMSSD, and NN50, LF, HF and Total power, as summarized in Table 4. Regarding the cross-correlation coefficients, we found the strong correlation (>0.8) in the dry and wet conditions regardless of the electrode types. In addition, no statistical differences were identified. On the other hand, in the immersion without movement condition, the signal from Ag/AgCl electrode indicated weak correlation (<0.5) whereas the signal from PLS and PLP showed the strong correlation higher than 0.9. Definitely, the correlation coefficients in the immersion with movement condition were significantly reduced. However, as can be shown in Table 4, we found PLS and PLP showed higher correlation of 0.51 and 0.45, respectively, than that of Ag/AgCl with 0.19, which was reduced by approximately 0.3. Moreover, the statistical differences were identified within each pair: Ag/AgCl *vs.* PLS and Ag/AgCl *vs.* PLP in the freshwater immersion with/without movement conditions. Thus, the signals obtained from PLS and PLP electrodes showed stronger correlation than the ones from Ag/AgCl electrode.

### 3.2. Under Saltwater Condition (Case-II): PLS vs. Ag/AgCl and PLP vs. Ag/AgCl

**Figure 10:**
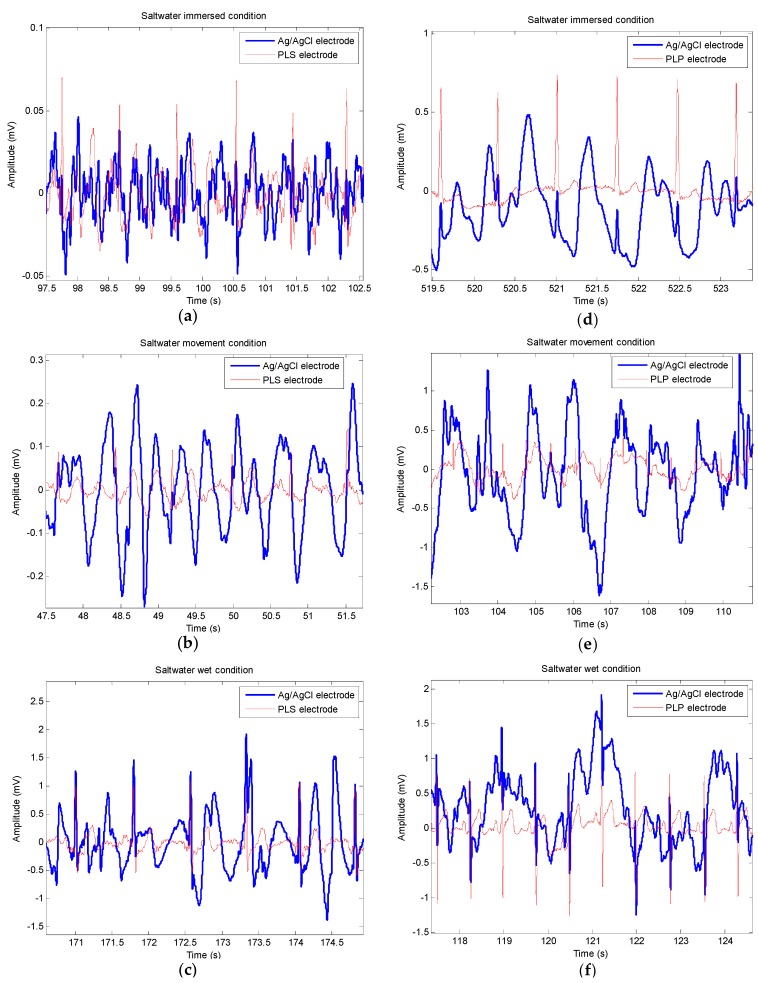
Example of electrocardiogram signals for each experimental condition. (**a**–**c**) Pencil lead solid-type electrode compared with Ag/AgCl electrode; (**d**–**f**) Pencil lead powder-type electrode compared with Ag/AgCl electrode; Saltwater immersed condition: (**a**,**d**) without movement; and (**b**,**e**) with movement; (**c,f**) Saltwater wet condition.

Figure 10a–c shows approximately 5-s ECG data measurements using PLS and Ag/AgCl electrodes under the saltwater immersion condition, followed by the movement condition and the wet condition, respectively. It is shown that the PLS electrodes provided better quality of ECG signals with clear defined R-waves in all three conditions. On the other hand, the ECG signals from Ag/AgCl were almost indiscernible. Similarly, Figure 10d through f show approximately 5-s ECG data measurements measured by PLP and Ag/AgCl electrodes under the same conditions. It is also shown that the PLP electrodes provided better quality of ECG signals in all three conditions.

Statistical results of temporal and spectral HRV indices obtained with Ag/AgCl, PLS, and PLP electrodes are summarized in Table 6, Table 7 and Table 8 for the saltwater immersion without movement, followed by immersion with movement, and the wet conditions, respectively. We identified statistical differences in the all conditions. More specifically, without movement, the statistical differences were identified when the Ag/AgCl *vs.* PLS was with RMSSD, LF and HF, and the Ag/AgCl *vs.* PLP was with SDNN, RMSSD, LF and HF as summarized in Table 6. With movement, the statistical differences were identified when the Ag/AgCl *vs.* PLS was with mean NN, SDNN, RMSSD, and total power, and the Ag/AgCl *vs.* PLP was with mean NN, SDNN, RMSSD, LF, HF, and total power as summarized in Table 7. In the saltwater wet condition, the significant statistical differences were identified when the Ag/AgCl *vs.* PLS was with SDNN, RMSSD, LF, HF, and LF/HF, and the Ag/AgCl *vs.* PLP is with SDNN, RMSSD, and HF as summarized in Table 8. Furthermore, we found the strong correlation (>0.8) under the wet condition regardless of the electrode types as shown in Table 8. On the other hand, in the immersion with/without movement conditions, the correlation coefficients from PLS and PLP showed higher values than that of Ag/AgCl, as shown in Table 6 and Table 7. The statistical differences were identified in the saltwater immersion with/without movement conditions.

### 3.3. Impedance Characterization

**Figure 11:**
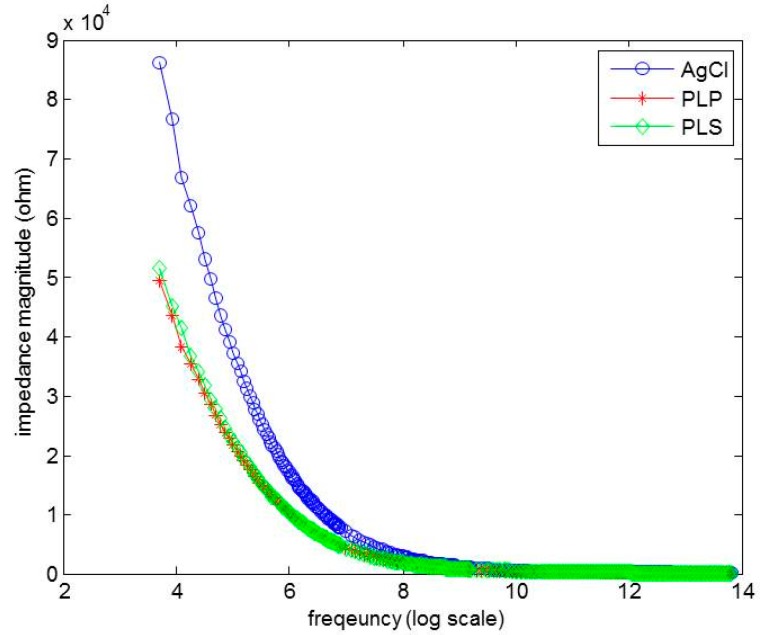
The impedance performance of Ag/AgCl, pencil lead powder-type, and pencil lead solid-type electrodes.

The electrode-to-skin contact impedances for PLP, PLS, and Ag/AgCl were measured and compared. The impedance magnitude |Z| values were plotted versus frequency in Figure 11, where the *x*-axis is a log scale. The PLP electrodes showed lower electrode-to-skin impedance magnitude followed by the PLS and Ag/AgCl. At 40 Hz, the electrode-skin impedance was 49.47 kΩ, 51.37 kΩ, and 86.12 kΩ for PLP, PLS, and Ag/AgCl, respectively, which shows that the PLP provides 1.03 and 1.74 times lower impedance than PLS and Ag/AgCl do.

## 4. Conclusions and Discussion

We have developed two types of dry electrodes from graphite material using pencil lead. Type-1 is the PLS electrode and Type-2, the PLP electrode. The performances of PLS and PLP electrodes have been compared with the commercial Ag/AgCl electrode, and the electrode-to-skin contact impedance have also been analyzed. The impedances of PLS and PLP are almost identical, and the values are smaller than those of the Ag/AgCl electrode. The comparison was analyzed for various conditions: dry surface, freshwater immersion with/without movement, post-freshwater (wet), saltwater immersion with/without movement, and post-saltwater (wet) conditions. We were able to capture all morphological ECG waveforms with our dry electrodes in all conditions.

When PLS and PLP were compared to Ag/AgCl, we identified significant statistical differences under all conditions, except for dry and freshwater wet conditions. Especially, under the saltwater wet condition after the immersion, R-peak from Ag/AgCl was observed, but the overall morphology was more unstable compared with that from PLS and PLP. Under the freshwater- and saltwater-immersion without movement conditions, we observed an R-wave amplitude reduction with PLS and PLP, but the signal had a good quality and the reduction was not severe enough to obscure the readability of the recordings. We believe that the graphite-based electrodes can be used for the new research area concerning underwater vital sign monitoring (including sea water).

## Figures and Tables

**Table 1:** Experimental protocol.

*Index*	Experiment-I: Ag/AgCl *vs.* PLS		Experiment-II: Ag/AgCl *vs.* PLP	
*(i)*	Dry Condition (5 min)		Dry Condition (5 min)	
	***Case-I: Freshwater***	***Case-II: Saltwater***	***Case-I: Freshwater***	***Case-II: Saltwater***
*(ii)*	Immersion without Movement (5 min)		Immersion without Movement (5 min)	
*(iii)*	Immersion with Movement (3 min)		Immersion with Movement (3 min)	
*(iv)*	Wet Condition (5 min)		Wet Condition (5 min)	

**Table 2:** Electrocardiogram signal quality—dry condition.

	Parameter	Ag/AgCl	PLS	*p*-Value	Ag/AgCl	PLP	*p*-Value
Temporal measures of HRV	meanNN (ms)	700.5 ± 89.6	700.1 ± 91.1	0.9816	723.4 ± 86.2	723.0 ± 83.8	0.9853
	SDNN (ms)	59.2 ± 22.5	55.8 ± 27.3	0.7819	54.2 ± 16.0	48.8 ± 16.3	0.4171
	RMSSD (ms)	58.7 ± 39.8	51.3 ± 42.4	0.7075	50.4 ± 27.9	38.3 ± 27.8	0.2986
	NN50	23.22 ± 19.44	21.77 ± 19.33	0.8764	28.83 ± 21.88	27.75 ± 23.31	0.9076
Spectral measures of HRV	LF (ms^2^)	1258 ± 783	1066 ± 757	0.6042	992 ± 760	895 ± 632	0.7371
	HF (ms^2^)	1391 ± 2003	701 ± 1287	0.3976	757 ± 812	498 ± 623	0.3914
	Total (ms^2^)	431562 ± 81172	429086 ± 79566	0.9487	450714 ± 101211	449358 ± 100911	0.9740
	LF/HF	3.66 ± 2.69	4.29 ± 2.54	0.6182	2.25 ± 1.71	3.61 ± 2.84	0.1685
Corr-Coef	*r* (unitless)	-	0.99 ± 0.005	-	-	0.99 ± 0.001	*-*

**Table 3:** Electrocardiogram signal quality—freshwater immersion without movement condition.

	Parameter	Ag/AgCl	PLS	*p*-Value	Ag/AgCl	PLP	*p*-Value
Temporal measures of HRV	meanNN (ms)	840.0 ± 155.6	828.0 ± 97.8	0.8358	778.0 ± 275.1	746.9 ± 119.1	0.4171
	SDNN (ms)	112.9 ± 64.0	53.5 ± 13.8	0.0223	225.4 ± 135.2	72.8 ± 27.6	0.0044
	RMSSD (ms)	144.9 ± 101.7	54.5 ± 20.0	0.0271	304.5 ± 189.0	75.9 ± 41.8	0.0027
	NN50	61.37 ± 39.28	43.0 ± 33.6	0.3318	162.33 ± 108.57	102.88 ± 99.19	0.2429
Spectral measures of HRV	LF (ms^2^)	3809 ± 3317	1105 ± 686	0.0404	11390 ± 15613	1514 ± 1238	0.0767
	HF (ms^2^)	7168 ± 7290	886 ± 600	0.0292	24366 ± 38574	1678 ± 2052	0.0971
	Total (ms^2^)	667061 ± 15484	638996 ± 15272	0.7205	584884 ± 181314	492856 ± 147666	0.2549
	LF/HF	1.03 ± 0.74	1.42 ± 0.66	0.2799	0.62 ± 0.29	1.47 ± 0.89	0.0154
Corr-Coef	*r* (unitless)	0.41 ± 0.23	0.96 ± 0.02	0.0000	0.41 ± 0.23	0.93 ± 0.04	0.0000

**Table 4:** Electrocardiogram signal quality—freshwater immersion with movement condition.

	Parameter	Ag/AgCl	PLS	*p*-Value	Ag/AgCl	PLP	*p*-Value
Temporal measures of HRV	meanNN(ms)	1130.0 ± 799.9	753.1 ± 171.5	0.0205	1146.3 ± 1055.1	728.0 ± 139.0	0.0145
	SDNN (ms)	754.4 ± 383.7	152.6 ± 70.1	0.0087	834.0 ± 620.1	122.2 ± 53.5	0.0060
	RMSSD (ms)	1010.2 ± 579.7	199.0 ± 103.1	0.0151	1109.0 ± 832.3	156.3 ± 72.8	0.0061
	NN50	117.4 ± 17.03	48.6 ± 32.02	0.0028	83.12 ± 51.48	28.62 ± 18.2	0.0136
Spectral measures of HRV	LF (ms^2^)	223079 ± 24173	4007 ± 3893	0.0773	168449 ± 189442	3319 ± 2135	0.0272
	HF (ms^2^)	354946 ± 44101	8967 ± 12222	0.1176	348641 ± 392893	6166 ± 4855	0.0272
	Total (ms^2^)	2336098 ± 1864	535985 ± 10881	0.0633	2268620 ± 2114	475750 ± 69503	0.0310
	LF/HF	0.72 ± 0.72	0.74 ± 0.61	0.9582	0.61 ± 0.22	0.80 ± 0.61	0.4278
Corr-Coef	*r* (unitless)	0.19 ± 0.11	0.51 ± 0.16	0.0000	0.19 ± 0.11	0.45 ± 0.21	0.0000

**Table 5:** Electrocardiogram signal quality—freshwater wet condition.

	Parameter	Ag/AgCl	PLS	*p*-Value	Ag/AgCl	PLP	*p*-Value
Temporal measures of HRV	meanNN(ms)	811.6 ± 100.6	810.8 ± 97.2	0.9670	794.6 ± 130.2	792.5 ± 116.9	0.9609
	SDNN (ms)	73.7 ± 26.6	69.4 ± 24.7	0.7767	84.2 ± 41.8	70.7 ± 24.9	0.4465
	RMSSD (ms)	70.4 ± 38.0	61.2 ± 21.6	0.6163	86.8 ± 62.5	63.7 ± 28.2	0.3563
	NN50	56.83 ± 46.69	55.16 ± 46.08	0.9516	62.12 ± 50.25	58.87 ± 50.14	0.8988
Spectral measures of HRV	LF (ms^2^)	2293 ± 2142	2182 ± 2098	0.9295	2344 ± 2406	2156 ± 1962	0.8660
	HF (ms^2^)	1722 ± 2032	819 ± 837	0.3377	1408 ± 1778	1306 ± 942	0.8888
	Total (ms^2^)	626275 ± 13981	621357 ± 13557	0.9518	585003 ± 147931	584516 ± 145797	0.9947
	LF/HF	2.44 ± 2.10	3.29 ± 2.32	0.5219	3.27 ± 3.57	3.01 ± 3.54	0.8840
Corr-Coef	*r* (unitless)	0.89 ± 0.01	0.88 ± 0.01	0.2551	0.89 ± 0.01	0.90 ± 0.01	0.2565

**Table 6:** Electrocardiogram signal quality—saltwater immersion without movement condition.

	Parameter	Ag/AgCl	PLS	*p*-Value	Ag/AgCl	PLP	*p*-Value
Temporal measures of HRV	meanNN (ms)	882 ± 162	823 ± 86	0.3275	841 ± 103	813 ± 110	0.4972
	SDNN (ms)	359 ± 383	130 ± 43	0.0776	313 ± 154	124 ± 82	0.0004
	RMSSD (ms)	428 ± 396	175 ± 60	0.0410	421 ± 210	158 ± 117	0.0004
	NN50	92.4 ± 73.5	62.5 ± 38.3	0.2903	246.2 ± 184.5	159.6 ± 157.5	0.1998
Spectral measures of HRV	LF (ms^2^)	21595 ± 31984	2845 ± 1473	0.0405	25621 ± 24694	5994 ± 7756	0.0087
	HF (ms^2^)	40166 ± 60509	6642 ± 5420	0.0480	43893 ± 39516	10705 ± 14515	0.0066
	Total (ms^2^)	753959 ± 34646	606370 ± 14328	0.2291	652235 ± 238080	557039 ± 206664	0.2688
	LF/HF	0.5562 ± 0.1238	0.5097 ± 0.1648	0.4850	0.5673 ± 0.1620	1.2010 ± 1.8080	0.2029
Corr-Coef	*r* (unitless)	0.57 ± 0.19	0.73 ± 0.10	0.0000	0.57 ± 0.19	0.69 ± 0.06	0.0000

**Table 7:** Electrocardiogram signal quality—saltwater immersion with movement condition.

	Parameter	Ag/AgCl	PLS	*p*-Value	Ag/AgCl	PLP	*p*-Value
Temporal measures of HRV	meanNN(ms)	1454 ± 223	938 ± 124	0.0020	1277 ± 355	851 ± 120	7.11e-04
	SDNN (ms)	1102 ± 315	497 ± 190	0.0054	872 ± 412	335 ± 243	7.92e-04
	RMSSD (ms)	1481 ± 309	677 ± 265	0.0023	1197 ± 558	448 ± 293	4.53e-04
	NN50	132.2 ± 41.2	117.6 ± 22.9	0.5072	171.8 ± 116.5	102.1 ± 58.5	0.0785
Spectral measures of HRV	LF (ms^2^)	296431 ± 35372	45736 ± 29809	0.1529	174843 ± 241251	26346 ± 30832	0.0459
	HF (ms^2^)	531535 ± 37302	146587 ± 10063	0.0564	439754 ± 499125	57801 ± 58326	0.0151
	Total (ms^2^)	2984351 ± 1396	1088667 ± 4357	0.0199	2459872 ± 20369	748602 ± 261051	0.0085
	LF/HF	0.4661 ± 0.2187	0.3257 ± 0.0298	0.1928	0.4170 ± 0.1328	0.4440 ± 0.1370	0.6284
Corr-Coef	*r* (unitless)	0.21 ± 0.14	0.51 ± 0.17	0.0000	0.21 ± 0.14	0.63 ± 0.13	0.0000

**Table 8:** Electrocardiogram signal quality—saltwater wet condition.

	Parameter	Ag/AgCl	PLS	*p*-Value	Ag/AgCl	PLP	*p*-Value
Temporal measures of HRV	meanNN (ms)	789 ± 60	756 ± 67	0.4320	848 ± 121	823 ± 114	0.5957
	SDNN (ms)	228 ± 89	60 ± 17.8	0.0032	173 ± 107	77.2 ± 37.4	0.0055
	RMSSD (ms)	297 ± 123	55.8 ± 28.5	0.0027	219 ± 142	86.2 ± 69.7	0.0059
	NN50	70.8 ± 44.7	44.4 ± 47.7	0.4028	82.5 ± 49.6	68.1 ± 57.1	0.5011
Spectral measures of HRV	LF (ms^2^)	12189 ± 8218	926 ± 568	0.0156	12170 ± 19375	1476 ± 1108	0.0584
	HF (ms^2^)	19837 ± 16445	633 ± 458	0.0311	16844 ± 25268	2123 ± 3183	0.0479
	Total (ms^2^)	589723 ± 85193	497650 ± 95412	0.1461	686362 ± 196078	620400 ± 186430	0.3881
	LF/HF	0.7470 ± 0.2407	1.8791 ± 0.9075	0.0272	1.0658 ± 0.7633	1.5443 ± 1.0340	0.1920
Corr-Coef	*r* (unitless)	0.97 ± 0.01	0.92 ± 0.008	0.2039	0.97 ± 0.01	0.98 ± 0.003	0.2951

## Abbreviations

The following abbreviations are used in this manuscript:

ECG or EKG	Electrocardiography
GPL	Graphite Pencil Lead
PLS	Pencil Lead Solid-type
PLP	Pencil Lead Powder-type
Ag/AgCl	Silver/SilverChloride
ANS	Autonomic Nervous System
HRV	Heart Rate Variability
DCS	Decompression Sickness
FFT	Fast Fourier Transform
PSD	Power Spectrum Density
PVCs	Premature Ventricular Contractions
PACs	Premature Atrial Contractions
meanNN	mean Normal-to-Normal interval
SDNN	Standard Deviation of all NN intervals
RMSSD	(square) Root of the Mean Squared Successive Differences of NN intervals
NN50	number of interval differences of successive NN intervals greater than 50 ms
LF	Low Frequency
HF	High Frequency
HR	Heart Rate
